# Retrospective Evaluation of the Most Frequently Observed Histological Changes in Duodenal and Rectal Mucosal Biopsies in Horses with Recurrent Colic

**DOI:** 10.3390/ani12243527

**Published:** 2022-12-13

**Authors:** Natalia Siwińska, Agnieszka Żak-Bochenek, Marzena Paszkowska, Maciej Karczewski, Dorota Długopolska, Wolfram Haider

**Affiliations:** 1 Department of Internal Medicine and Clinic of Diseases of Horses, Dogs and Cats, Faculty of Veterinary Medicine, Wroclaw University of Environmental and Life Sciences, 50-375 Wrocław, Poland; 2 Department of Immunology, Pathophysiology and Veterinary Preventive Medicine, Wroclaw University of Environmental and Life Sciences, 50-375 Wrocław, Poland; 3 Vetlab Polish Veterinary Laboratories, 52-017 Wrocław, Poland; 4 Department of Applied Mathematics, Faculty of Environmental Engineering and Geodesy, Wrocław University of Environmental and Life Sciences, 50-375 Wrocław, Poland; 5 Faculty of Veterinary Medicine, Wroclaw University of Environmental and Life Sciences, 50-375 Wrocław, Poland; 6 Institut für Tierpathologie, 14163 Berlin, Germany

**Keywords:** biopsy, equine, colic, inflammation, cellular infiltration

## Abstract

**Simple Summary:**

Colic, a condition affecting the digestive tract of horses, manifests itself in severe pain and may be life-threatening. Recurrent colic is usually caused by ongoing, chronic inflammatory process (and may be defied as: ≥3 episodes of colic within a 6-month period, with at least 48 h between colic episodes), associated with a chronic process, often of an inflammatory nature. Multiple causes of recurrent colic may exist, including, for an improper diet, management changes, stress, or parasites infestation. During the diagnostic process, a noninvasive test in the form of taking a biopsy of the duodenal or rectal mucosa proves to be useful, but its usefulness is limited only to diffuse processes. The study presented here depicts a retrospective analysis of the histopathological findings of samples taken from 77 horses with recurrent colic, focusing on cellular infiltration, fibrosis, and erosions. All samples from the duodenum showed the presence of leukocytes infiltrates in the mucosal lamina propria, of which almost 70% were diffuse infiltrates. The most frequently observed cellular infiltration was a moderate infiltration consisting of lymphocytes and plasma cells. More than one-fourth of the horses were also found to have shortened intestinal villi. Similarly, in biopsies from the rectum, cellular infiltration was observed in all horses in this section, which consisted of a mixed population of plasma cells, lymphocytes, and eosinophilia. Analysis of the inflammatory lesions present may help in understanding the pathogenesis of chronic colic in horses.

**Abstract:**

Colic, a condition affecting the gastrointestinal tract of horses, manifests as severe pain and may be a life-threatening condition. It is possible to distinguish between an acute, disposable process, as well as recurrent colic symptoms (abdominal pain) caused by an ongoing chronic inflammatory process. This paper presents a retrospective analysis of the histopathological findings of duodenal and rectal samples taken from horses with recurrent colic, with the aim to determine the frequency and extent of inflammation. The samples, i.e., duodenal biopsy (60 samples) and rectal biopsy (17 samples), were taken from 77 horses showing recurrent colic symptoms. Histopathological examination included staining with hematoxylin and eosin. The examination included evaluation of the superficial epithelium, mucosal lamina propria, and submucosa. All samples from the duodenum and rectum showed the presence of leukocyte infiltration in the mucosal lamina propria. The most frequently observed cellular infiltration was a moderate infiltration consisting of lymphocytes and plasma cells in duodenum and mixed populations of plasma cells, lymphocytes, and eosinophilia in the rectum. Mott cells were also noted among the inflammatory infiltrates. More than one-fourth of the horses were found to have shortened intestinal villi. The results presented here showed the involvement of inflammation in the course of recurrent colic, which can be both its cause (by impairing motility and absorption) and its effect (as a result of obstruction or ischemia).

## 1. Introduction

Colic is one of the most common problems in horses and causes a significant amount of euthanasia or death in these species [1,2,3]. Recurrent colic is a separate serious issue, mainly with mild clinical signs (pawing, turning the head to the flank, lying down without rolling or sweating), in which it is often difficult to find a clear cause of the problem [4]. For purposes of the present study, recurrent colic was defined as ≥3 episodes of colic within a 6-month period, with at least 48 h between colic episodes [1]. As is well known, there are many predisposing factors for recurrent colic, and these include, among others: incorrect diet, management changes, stress, parasites infestation, and behavioral disturbances [5,6]. Research published so far on this matter in horses would mainly focus on the causes and survival of animals [7].

The occurrence of changes in the intestinal wall, like inflammation, neoplastic infiltration, or fibrosis may be the cause of recurrent colic, as well as a consequence of another reason for colic [7,8]. Infiltration of different cell populations (eosinophils, plasma cells, lymphocytes, basophils, and macrophages) in mucosa and submucosa can cause gastrointestinal tract dysfunction due to inflammation and malabsorption [8]. In vitro studies have shown that inflammation of the small intestine, even of a minor degree, has a debilitating effect on intestinal contractility while impairing motility [9]. In the case of intestine inflammation, multiple components of the inflammatory response lead to mucosal hypoxia [10]. The complexity of this issue often makes it impossible to determine which factor occurred first: colic or inflammatory bowel disease. In horses, inflammatory changes observed in both duodenal and rectal biopsies showed a correlation with weight loss, which may also suggest digestive and/or absorption disorders [8,11]. The long-term diagnostic process often does not use analysis of intestinal biopsies, opting mainly for noninvasive investigations. Field-feasible biopsies of the duodenum and rectum do not always provide sufficient data, especially for focal processes. The decision about intestinal biopsy in the case of focal condition is most often made during a laparotomy. The choice to undergo surgery is usually made in the case of acute colic signs unresponsive to conservative treatment. However, in these cases, intestines already show inflammatory or necrotic changes. Another problem in horses with chronic colic is the “blind” treatment with steroids by the primary doctor, often noted by the authors, which can alter the picture of intestinal inflammation if a biopsy is taken. The issue of histological changes in horses has been described in two broad publications—the first examined 116 horses with gastrointestinal problems (weight loss, diarrhea, lack of appetite, and rarely colic) and the second, which described 66 cases of recurrent colic in horses, with reference to the clinical picture [7,12]. The authors first extended the observation by comparing the changes and occurrence of hypoproteinemia.

The aim of the study was to determine the type and severity of histopathological changes, with particular emphasis on cellular infiltration, in the mucosa of the duodenum and rectum in horses with mild signs of recurrent colic and comparing the changes with the total protein and albumin values.

## 2. Materials and Methods

The study was conducted on mucosa samples taken from 77 mix breed horses (49 males, 28 females) with average ages 11.7 +/− 6.4 years, admitted in the years 2018–2022 with signs of mild recurrent colic. The tested horses came from Poland, the dominant breed of the tested animals were horses of noble breed, including thoroughbreds, Greater Poland, Lesser Poland, and mixed breeds. The tested animals were both unused (retirement, injury) and used (recreation, sport) animals. The animals were fed hay as roughage and oats or commercial fodder as concentrated food. In the conducted study, mild colic was defined in accordance with the available literature [4]. For purposes of the present study, recurrent colic was defined as ≥3 episodes of colic within a 6-month period, with at least 48 h between colic episodes [1]. Horses were referred for further diagnostics after a different number of colic episodes, usually from 3 to 6 episodes. A series of diagnostic tests were carried out in all animals to find the cause of recurrent colic, i.e., full clinical exam, blood work, fecal exam, rectal palpation, transabdominal and transrectal ultrasonography, gastroscopy, and peritoneal fluid analysis. Analysis of total protein and serum albumin was carried out using the colorimetric method and analysis with Beckman Coulter AU 680, using copper ions and Bromocresol Green, respectively. According to the laboratory’s guidelines, the following were considered reference standards: 55–72 g/L and 25–44 g/L for total protein and albumins, respectively. In most cases, diagnostic tests were performed during the same diagnostic visit. Some horses had previously been diagnosed by other doctors and were referred for biopsy only. The presence of internal parasites was also excluded on the basis of fecal examination (flotation, sedimentation, and the McMaster method). None of the animals underwent surgery. Most duodenal and rectal biopsies were taken after the episode of colic symptoms was resolved (day to week after episode). In the samples taken, 60 sections were taken from the duodenal mucosa, which were collected using biopsy forceps through the working canal of the gastroscope. These fragments were collected under sedation (Detomidine 0.01–0.02 mg/kg IV (Domosedam 10 mg/mL, Orion Corporation, Espoo, Finland) and Butophanol 0.01–0.02 mg/kg IV (Morphasol 10 mg/mL, aniMedica BmbH, Frankfurt, Germany)) during gastroscopic examination, preceded by a 14-h fast [13]. During the gastroscopic examination, the best site for the biopsy was selected. In the case of horses with visible macroscopic changes (change in color of the mucosa, change in mucosal surface, visible proliferative changes), the biopsy was taken from these places and from the border with healthy tissue. For horses with no lesions, the best available site for biopsy was selected. The biopsy was considered successful if an adequate fragment (filling the entire biopsy forceps) was obtained and bleeding was visible from the biopsy site. Seventeen biopsies were obtained from the mucosa of the rectum using mare uterine biopsy forceps. The procedure was performed after pharmacological sedation (Detomidine 0.01–0.02 mg/kg IV (Domosedam 10 mg/mL, Orion Corporation, Espoo, Finland) and Butophanol 0.01–0.02 mg/kg IV (Morphasol 10 mg/mL, aniMedica BmbH, Frankfurt, Germany)). After the feces were removed from the rectum, a finger biopsy was taken at 10 o’clock and 2 o’clock [13]. The biopsy was considered successful if an adequate fragment (filling the entire biopsy forceps) was obtained. Each patient had 2 to 4 sections of mucosa, which were then placed in a container with 4% buffered formalin for 24 h. The collected and fixed biopsies were sent to the Institut für Tierpathologie, Berlin, Germany for histological examination, which was performed by a certified veterinary pathologist experienced in evaluating intestinal mucosa biopsies in horses (WH). Biopsies were embedded in paraffin, then cut into 2 µm thick sections and stained with hematoxylin and eosin. Histological examination was performed using Olympus BX53 optical microscope (Olympus, Tokyo, Japan) equipped with a digital Olympus ColorView IIIu camera (Olympus, Tokyo, Japan) and analyzed under 50×, 100×, 200×, and 400× magnification.

In the histological examination, the structure of the superficial epithelium, lamina propria, and the submucosa of the mucosa were assessed sequentially. In the superficial epithelium, its profile, exfoliated cells, the presence of necrosis, the presence of erosions, mucus, fibrin, and eosinophilia, as well as the appearance of intestinal villi and goblet cells, were assessed. In the lamina propria, the presence and type of infiltration (diffuse/focal; non/mild/moderate/marked; low/high) and the type of cells included in the infiltration (lymphocytes, plasma cells, Mott cells, eosinophils, neutrophils, and macrophages) were assessed. Additionally, in this section, the fibrosis, the presence of edema, and the abundance of Brunner’s glands were assessed, based on the available literature [14]. In the submucosa, as in the lamina propria, the type of infiltration and its cellular composition were assessed. Additionally, the presence of lymph nodes, dilatation of lymph vessels, and blood vessel congestion were also assessed. The obtained results were analyzed by assessing how many horses had a given change and presenting it as a percentage. To perform statistical calculations of correlation, the scale of changes in the intestines was changed from descriptive to numerical: 0—non, 1—mild, 2—moderate, and 3—marked. 

*Statistical analysis:* Descriptive statistics were presented as the mean and standard deviation. Differences between groups were analyzed with the independent samples *t*-test and Mann–Whitney *U* test. The correlations were analyzed using the Kruskal–Wallis test [15,16]. Statistical results were considered significant when the *p*-value was below 0.05.

## 3. Results

### 3.1. Duodenal Mucosal Samples

#### 3.1.1. Superficial Epithelium

**Figure 1:**
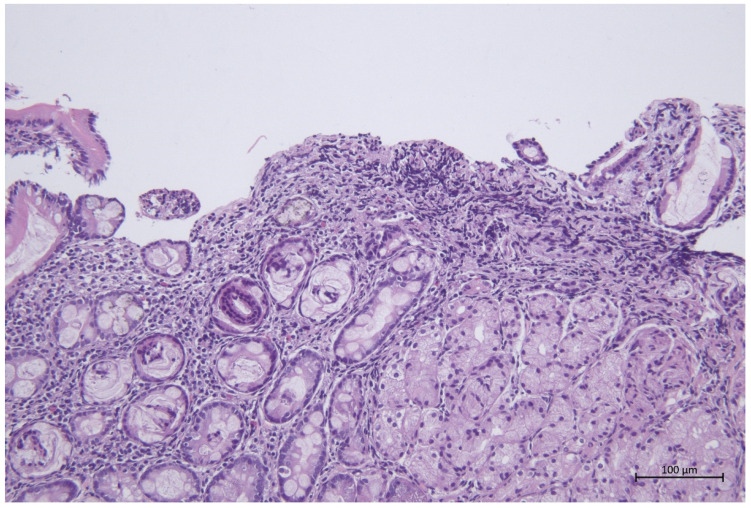
Erosive duodenitis, 200× magnification.

Epithelial hypoplasia was observed in 3 out of 60 horses (5%) and in 9 (15%) of its structure disorder. The exfoliation of epithelial cells was observed in 7 horses (11.6%), necrotic changes in 2/60 (3.3%), and erosions in 4/60 (6.6%) of the tested samples (Figure 1). The presence of mucus was observed in 5/60 (8.3%). Fibrosis was observed in 2/60 (3.3%) of the samples. The infiltration of eosinophils was observed only in 2/60 (3.3%) of the examined sections of the duodenum. Shortening of the intestinal villi was the most frequently observed phenomenon and occurred in 16/60 (26.7%) of cases. In 2/60 (3.3%), the villi were thickened, and in 2/60 (3.3%) of horses, they were partially fused. Few goblet cells were also observed in 11/60 (18.3%) of the horses.

#### 3.1.2. Lamina Propria

**Figure 2:**
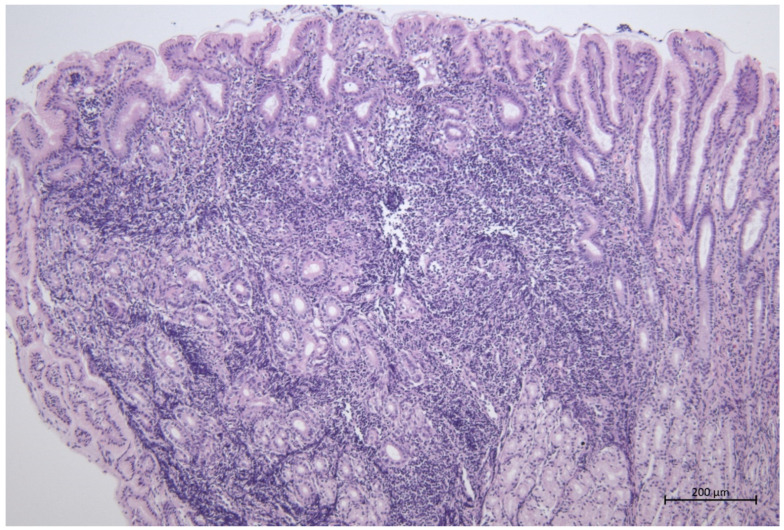
Marked lymphocytic duodenitis, magnification 100×.

**Figure 3:**
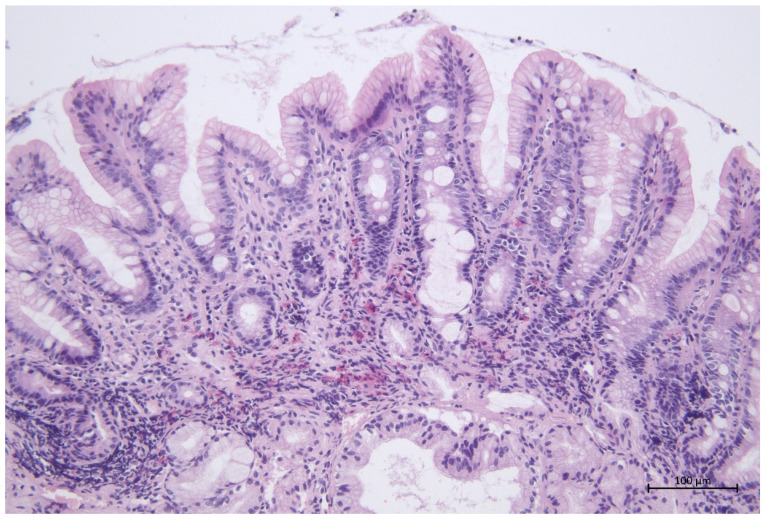
Eosinophilic duodenitis, 200× magnification.

**Figure 4:**
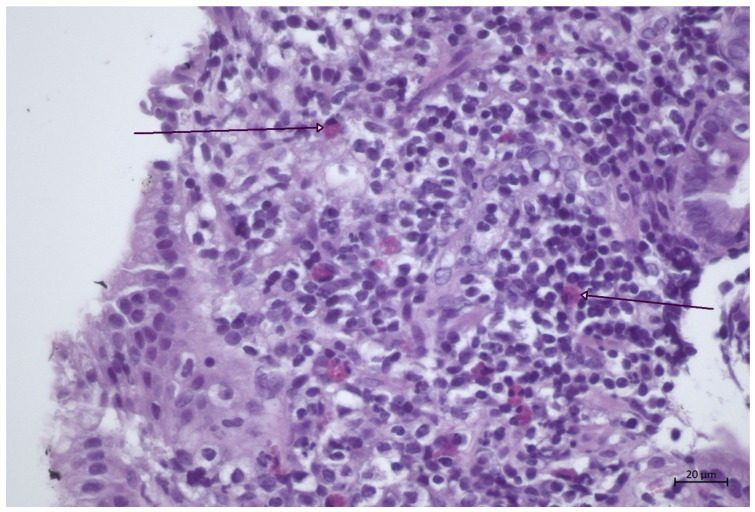
Histopathological image of a horse diagnosed with moderate subacute to chronic non-ulcerative duodenitis. Mott’s cells are marked with an arrow. Magnification 600×.

**Figure 5:**
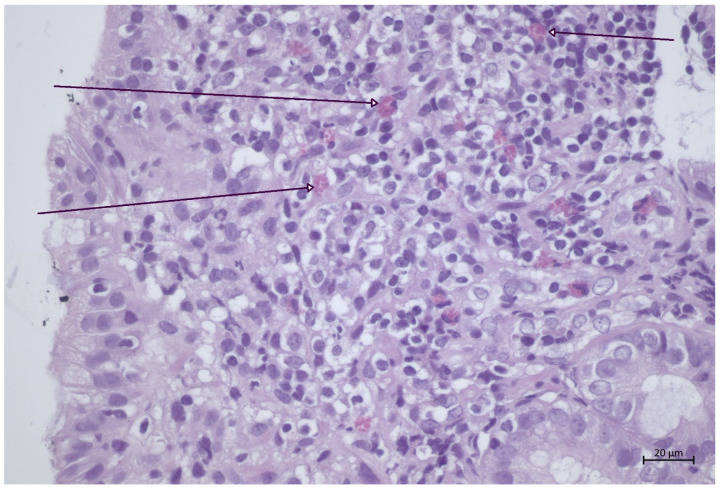
Histopathological image of a horse diagnosed with moderate subacute to chronic non-ulcerative duodenitis. Mott’s cells are marked with an arrow. Magnification 600×.

Cell infiltration of the lympho- and myeloblastic origin cells was present in all samples from all horses tested. Most (41/60) (68.3%) of the horses showed diffuse inflammatory infiltration, and only 5/60 (8.3%) had a focal infiltration. Some (16/60) (26.7%) of the samples showed a mild inflammatory infiltrate, 19/60 (31.6%) moderate, and 5/60 (8.3%) marked. The results regarding the type of cells forming inflammatory infiltrate are presented in Table 1. The most frequently observed cellular infiltration was a moderate infiltration consisting of lymphocytes and plasma cells (Figure 2); these cells were the most frequently observed in the tested population. The infiltration of neutrophils and eosinophils was observed in more than half of the horses (Figure 3). In 3/60 (5%), the presence of Mott cells in the lamina propria of the mucosa was confirmed (Figure 4 and Figure 5). Fibrosis in the duodenal lamina propria was seen in 6/60 (10%) of horses. Lamina swelling was observed in 15/60 (25%) of the examined patients, and abundant Bruner’s glands were present in 16/60 (26.7%) of the examined patients.

#### 3.1.3. Submucosa

Cellular infiltration in this part of the intestinal wall was detached in 5/60 (8.3%), half of which was assessed as moderate and the others as marked. Cellular infiltration in this part of the duodenum was less frequently observed. The results regarding the type of cells forming the inflammatory infiltrate are presented in Table 2. Blood vessel congestion was observed in 4/60 (6.6%) of horses, lymphatic vasodilation in 3/60 (5%), and lymphoid formation in 4/60(6.6%).

From additional observations in this region of the intestines, the following were observed in individual horses: focal hemorrhage in the duodenal wall (one horse), extensive degeneration of all layers of the intestinal wall (one horse), and mitotic figures in the lamina propria (one horse).

### 3.2. Rectal Mucosal Samples

#### 3.2.1. Superficial Epithelium

An irregular profile was observed in 2/17 (11.6%) of the samples and in 2/17 (11.6%) thin cylindrical cells in this area. Erosions were observed in 7/17 (41.2%) of the examined patients. In 6/17 (35.3%) of the horses, numerous amounts of goblet cells were seen in this area, and in 2/17 (11.6%), very numerous.

#### 3.2.2. Lamina Propria

Oedema of lamina propria was noticed in 4/17 (23.5%) of the samples. Cellular infiltration in this part was visible in all the examined patients—in 13/17 (76.8%)%, it was graded as moderate, in 4/17 (23.5%), as marked, in 5/17 (29.4%), it was diffuse, and in the remaining patients, local. The results regarding the type of cells forming inflammatory infiltrate are presented in Table 3. Cellular infiltration was observed in all horses in this section, which consisted of a mixed population of plasma cells, lymphocytes, and eosinophilia. Infiltration with an additional neutrophil component was observed in only half of the subjects.

#### 3.2.3. Submucosa

Dilated lymphatic vessels were observed in 7/17 (41.2%) of the subjects, and in 4/17 (23.5%), 0% lymphatic papules were present. Only one horse had hyperemia of the lamina propria. The infiltration of inflammatory cells in the lamina propria was observed in 15/17 (88.2%) of the horses. The results regarding the type of cells forming inflammatory infiltrate are presented in Table 4. Cellular infiltration in this section consisted of a mixed population of plasma cells, lymphocytes, and eosinophilia. Infiltration with an additional neutrophil component was observed in 6/17 (35.3%) of the samples.

### 3.3. Total Protein and Albumin Analysis

The levels of albumin and total protein in the serum of the tested horses are shown in Table 5, assuming reference standards of 25–44 g/L for albumin, hypoalbuminemia occurred in five horses, and assuming reference norms of 55–72 g/L for the total protein, hypoproteinemia was found in three horses. There were no correlations between the intensity of infiltration, the type of infiltration, and other observed changes and the values of serum albumin and total protein.

## 4. Discussion

The occurrence of histopathological changes, the type and extent of the inflammatory infiltration in the intestinal wall in horses with colic symptoms are still an important element of the discussion. The most interesting are the changes detected during duodenal or rectal biopsy, which are less invasive than a full intestinal wall biopsy and are feasible even in the field. In the presented study, the authors present very precisely the changes observed in the samples taken from the duodenum and rectum. In equine veterinary practice, we have few opportunities to take intestinal biopsies. Samples of the duodenum require a duodenoscopy procedure and include only the proximal portion of the duodenum. A diagnosis may be obtained in up to 20% of cases based on histopathological examination of duodenal biopsies [17]. Due to their relatively noninvasive nature, this method may be used as a preliminary approach. The second most common biopsy method is rectal biopsy. According to a study by Linberg et al. and Schumacher et al., 2000, in 50–52% of horses diagnosed for intestinal diseases, this biopsy revealed pathological changes [8,12]. However, the diagnosis of LPE or EE was unlikely to be supported by rectal biopsy [8,12]. According to available studies, rectal biopsies from 60 out of 116 horses with clinical signs of intestinal disease showed pathological changes. Similar changes were not found during histological examination of rectal biopsies from 30 clinically healthy horses [12].

One of the most common and interesting phenomena observed in the studied samples of the intestinal mucosa was cellular infiltration, which was observed in all horses in duodenal and rectal lamina propria. The infiltration of inflammatory cells in the submucosa was observed in 88.2% of horses in the rectum samples and only in 8.3% of the duodenal samples. The infiltration in part of the superficial epithelium was the least frequent, and it concerned only the duodenum in 5% of the examined samples. In the studies of Stewart et al., the presence of inflammatory infiltration was demonstrated only in 55% of horses with colic symptoms [7]. Such a high result in our research may result from different populations of the tested horses. Additionally, in the Stewart tests, the samples were collected in a less consistent manner than in our tests (horses with gastrointestinal pathologies), which could have influenced the results. The most common component of the infiltration in the collected samples was lymphocytic-plasmocytic, which is consistent with previously published studies [7]. Interestingly, in dogs, it is the most common infiltration consisting of lymphocytic and plasmocytic cells [18,19]. This may indicate that it is the most common component of the inflammatory infiltrate also in horses and may be associated with recurrent colic symptoms. The presence of lymphocyte–plasma cell infiltration into the lamina propria has been recognized as an essential diagnostic feature of chronic inflammatory bowel disease in humans [20]. Lymphocytic-plasmocytic enteritis (LPE) is characterized by infiltration of lymphocytes and plasma cells at the level of the lamina propria [21,22]. However, recent studies showed that lymphocytes and plasma cells may be normally present in the rectal lamina propria of healthy horses [12]. Other reports show that the presence of an excessive amount of these cells may be a confirmation of the pathological condition associated with the infiltration of these cells [12]. Nevertheless, an increase in the number of lymphocytes and plasma cells do not warrant a definitive diagnosis. Studies correlating rectal biopsy findings with equine intestinal disorders suggest that lymphoid and plasma cells may be present in rectal biopsy in many intestinal diseases (LPE, cyanosis, granulomatous disease, and gastrointestinal lymphoma). They therefore represent a nonspecific pattern of hyperactivity in the intestinal mucosa, rather than a specific disease entity [12,22]. It is also possible that colicky horses may have cellular infiltrates associated with intestinal inflammation during an episode of colic. In dogs, the presence of LPE is also associated with the risk of neoplastic lesions, such as extensive intestinal lymphocytes [19,20]. However, it is believed that LPE infiltration may be a pre-lymphoma state in dogs, although this relationship has not been studied in horses [12]. The next most common population of cells constituting an infiltration in the studied samples were eosinophils (they were present in all rectal samples in lamina propria and 82.4% in rectal submucosa, more than half of the duodenum samples in lamina propria, and very few in duodenal submucosa) and neutrophils (they were found in more than half of the duodenum samples in lamina propria and very few in submucosa and in half of the rectal lamina propria and in 29.4% of rectal submucosa). According to the available literature, an infiltrate consisting of eosinophilia may suggest the presence of eosinophilic enteritis (EE). It is characterized by an infiltration of inflammatory cells, dominated by eosinophils at the level of the intestinal mucosa [21,22]. Eosinophilic infiltration of the lamina propria and rectal submucosa can also be observed in healthy horses [11,12]. A study by Rotting et al., focusing on assessing the prevalence of eosinophils in particular segments of the digestive tract of healthy horses, showed eosinophilic infiltrates predominating in the lamina propria in the region between the muscularis mucosae and the base of the villi (for the small intestine) and in the lower one-half of the section between the muscularis mucosae and the intestinal lumen (rectum) [23]. However, both sections showed the lowest number of eosinophils, in contrast to the colon. Eosinophilic infiltration was also diagnosed as a cause of IBD in 11 horses in a study by Scott et al. [24]. Similarly, neutrophils may be present within plaques of the mucosa proper but should not be located in the superficial epithelium or crypts and are always considered pathological in these places [12,22,25]. The least frequently observed infiltrate was that suggesting granulomatous enteritis (characterized by lymphoid and macrophage infiltration at the level of the mucosal lamina propria with variable numbers of plasma cells and giant cells), which is in line with the latest research data, which has shown that infiltration of these cells is very rare in horses [11,26]. In idiopathic bowel disease, the lamina propria and submucosa of the large and small intestines were diffusely infiltrated with eosinophils and other inflammatory cells [8]. Mott cells have the “cluster of grapes” appearance of plasma cell cytoplasm filled with immunoglobulin inclusions (Russell bodies). Russell bodies are formed as a result of the cellular response to excessive stimulation of plasma cells, leading to the accumulation of abundant, undegradable, condensed immunoglobulins. Mott cells may be seen in reactive plasmacytosis, such as chronic inflammation, autoimmune diseases, and hematopoietic tumors with plasmocytic differentiation, such as lymphoma, plasmacytoma, or lymphoplasmacytic lymphoma [27]. They had moderate infiltration for 5% of the duodenal sections and 11.8% of the rectal sections in the samples presented in this study. According to the literature, their presence in the gastrointestinal mucosa suggests a benign inflammatory process [27]. The presence of these cells has not yet been described in the analysis of intestinal biopsies from horses; therefore, their involvement in intestinal pathological processes in this species is not well understood. Their presence may indicate increased activity of the mucosal response and secretory immunoglobulin A production in the cases of inflammatory bowel disease in horses. Other changes observed in the tested samples include fibrosis, erosions, and shortening of the intestinal villi. According to the available literature, intestinal lesions are conditions that may be associated with chronic inflammation in horses [7,28]. Fibrosis was observed in the duodenum, especially in the superficial epithelium and in the lamina propria. Fibrosis is a chronic and progressive process characterized by an excessive build-up of extracellular matrix (ECM) components such as collagen. It can be the result of chronic inflammation or a way to repair damaged tissue [29]. Occurring in the intestines, it is considered a frequent complication, e.g., enteropathy, including inflammatory bowel disease (IBD), neoplastic, or postoperative changes. Due to the location of the fibrosis in the examined horses, they may indicate inflammation, especially due to cellular infiltration, as well as other types of damage. However, it will not be possible to confirm the reason. In the examined horses, erosions were observed only in the superficial epithelium layer, mainly in the rectum (41.2%) but also in the duodenum (6.6%). The presence of erosions and ulcers diagnosed microscopically on a histological examination, such as fibrosis, may also be an inflammatory phenomenon. Inflammation is caused and sustained by immune cell infiltration in the intestinal mucosa, which determines tissue damage characterized by the loss of epithelial cells and degradation of the extracellular matrix in the lamina propria, leading to ulceration [29,30]. As it is known, the rectum in horses is more susceptible to the occurrence of reactions in its area, which confirms the frequent occurrence of cellular inflammatory infiltration, which also creates some diagnostic difficulties. The changes also affected the superficial layer of the mucosa, which is also the most susceptible to damage. Changes in the intestinal villi were a frequently observed phenomenon in the duodenum and concerned as much as 26.7% of the tested horses. These changes may confirm the presence of inflammation in this area, most often on an immunological basis. Shortening (atrophy) of intestinal villi is a phenomenon observed in humans in connection with inflammation of the intestines. It can occur in the course of any immune-mediated inflammation. Villi may be blunted and shortened or appear atrophic when the lamina propria is infiltrated by different types of cells, such as macrophages, plasma cells, or lymphocytes [30]. The shortening of the villi and the consequent reduction in absorption area will result in weight loss. Damage to cellular barriers on the surface of the villi can also expose them to pathogen migration. The abundant presence of Goblet cells in the superficial epithelium was observed in the rectum, much less and in a smaller number of animals in the duodenum. According to the available knowledge, goblet cells are an integral part of the organ epithelial barrier and produce mucin products and play an important role in the immune response to mucosal antigens (e.g., parasites) [31]. The observed congestion of blood in the vessels may result from the formation of blood clots, which is a consequence of the release of inflammatory mediators from damaged epithelial and/or endothelial cells (activation of the antifibrinolytic coagulation cascade) [32,33].

Gastrointestinal pathologies in horses often present with significant hypoalbuminemia and hypoproteinemia. It can result from both absorption disorders and protein-loosening enteropathies (such as infiltrative enteritis). Analyzing the results obtained from the studied horses, no significant correlations were found between the values of total protein and serum albumin. In further investigations, it may be necessary to introduce a more sophisticated determination of the degree of changes than just mild/moderate, etc. A similar result was obtained in an investigation by Boshuizen et al., 2018, which also found no relationship between either duodenal or rectal biopsy findings or blood test results, such as albumin or total protein [11].We cannot make conclusions regarding the overall role of inflammation in recurrent colic. According to previous studies, it is difficult to make a definite diagnosis based on the result of a rectal or duodenal biopsy alone (or both at once) [7]. The location of the biopsy site is also important in making the diagnosis. Studies show that biopsies taken from the duodenum or rectum very often show inflammation while they give little data on the possible presence of ischemia or a neoplastic process [7]. However, in a study by Stewart et al., few individuals showed the presence of inflammation in biopsies of the duodenum (4/36 horses) and rectum (3/36 horses) [7]. Intestinal biopsy collections, whether mucosal or whole-wall biopsies, are always associated with the risk of complications. These include, for example, septic peritonitis, bleeding, and ulceration. However, mucosal biopsy, taken in the field, without the need for general anesthesia, is associated with fewer risks, so it is more often chosen by practitioners. To increase the quality of this investigation, it would have been more valuable to analyze whole-wall biopsies, but this was not feasible. By performing a noninvasive biopsy, we avoid the manipulation of the intestines that occurs during laparoscopy or laparotomy. Studies have shown that intraoperative intestinal manipulation causes an increase in neutrophilic inflammation, as well as in the migration of eosinophils toward the intestinal lumen [34]. The colic itself, depending on the cause (gasification, displacement, and hypoxia) and the duration of the pathology, may be the cause of the appearance of histological changes in the intestines. This is a complication when examining biopsies of the mucosa from horses with colic symptoms and may cause an overdiagnosis of IBD. We suggest that histologic examination of rectal and duodenal biopsies is probably not a sensitive diagnostic test for detecting cellular infiltrates in tissue layers other than the mucosa and may be nondiagnostic in the case of cellular infiltrates that do not correspond to the location of the biopsy (i.e., if the biopsy is performed away from the lesion site). Previous studies have shown that in only 3 of 66 horses was it possible to make a diagnosis based on the results obtained from rectal biopsy [7]. The study did not compare the changes in the duodenum and the rectum from the samples obtained from the same individual. There was no need for both biopsies in the animals referred for the study; however, the possibility of comparing the duodenal and rectal biopsy would further enable the determination of whether the process is diffuse and involves both the small and large intestine or localized. Most often, rectal biopsy was due to the lack of access of the gastroscope or biopsy forceps to the working canal or the inability to enter the duodenum during gastroscopy. The results obtained from ringing horses were not compared with the results obtained in healthy horses. This was due to the inability to have a controlled population (e.g., horses without gastrointestinal disease or horses that had only one acute episode of colic). Recognizing these limitations, the high prevalence of inflammatory disease and the histological features noted (fibrosis and proliferation-hypertrophy) in the present study that may be the result of chronic inflammation should prompt future studies to investigate the role of intestinal inflammation in horses with recurrent colic. Histopathological examination of samples taken from horse intestines requires considerable experience on the part of the assessor. In the future, the preparation of a numerical scale assessing the degree of cellular infiltration compared to healthy horses, analogous to the scale prepared for dogs and cats, may prove to be a useful diagnostic parameter [35].

## 5. Conclusions

In conclusion, in the cases of recurrent colic, histopathological examination of duodenal and rectal sections most often indicated the presence of inflammatory infiltrates and shortened intestinal villi in the duodenum. The study was the first to demonstrate the involvement of Mott cells in the course of inflammation of the gastrointestinal tract in horses, which is an interesting issue for further research.

## Figures and Tables

**Table 1:** Table showing the type of cellular infiltration in the lamina propria of the duodenum, along with its degree, number of horses, and % distribution in the studied horse population.

Infiltration Degree	Cell Type					
	Lymphocytes	Plasma Cells	Neutrophils	Eosinophils	Macrophages	Mott Cells
mild	9 (15%)	11 (18.3%)	28 (46.6%)	33 (55%)	-	-
moderate	40 (66.6%)	40 (66.6%)	5 (8.3%)	1 (1.6%)	2 (3.3%)	3 (5%)
marked	9 (15%)	8 (13.3%)	2 (3.3%)	4 (6.6%)	-	-
SUM	58 (96.6%)	59 (98.2%)	35 (58.2%)	38 (62.3%)	2 (3.3%)	3 (5%)

Note: A significant number of horses showed infiltration composed of more than one type of cell, so, although the infiltration concerned all horses from the study population, the sum is not equal to 100%.

**Table 2:** Table showing the type of cellular infiltration in the submucosa of the duodenum, along with its degree, number of horses (*n* = 60), and % distribution in the studied horse population.

Infiltration Degree	Lymphocytes	Plasma Cells	Neutrophils	Eosinophils
mild	1 (1.6%)	-	1 (1.6%)	4 (6.6%)
moderate	3 (5%)	3 (5%)	1 (1.6%)	1 (1.6%)
marked	-	1 (1.6%)	-	-
SUM	4 (6.6%)	4 (6.6%)	2 (3.2%)	5 (8.2%)

Note: A significant number of the horses showed infiltration composed of more than one type of cell, so, although the infiltration concerned all horses from the study population, the sum is not equal to 100%.

**Table 3:** Table showing the type of cellular infiltration in the lamina propria of the rectum, along with its degree, number of horses (*n* = 17), and % distribution in the studied horse population.

Infiltration Degree	Lymphocytes	Plasma Cells	Neutrophils	Eosinophils	Mott Cells
mild	-	-	8 (47%)	3 (17.6%)	-
moderate	14 (82.3%)	14 (82.3%)	-	14 (82.3%)	2 (11.8%)
marked	3 (17.6%)	3 (17.6%)	-	-	-
SUM	17 (100%)	17 (100%)	8 (47%)	17 (100%)	2 (11.8%)

Note: A significant number of horses showed infiltration composed of more than one type of cells, so, although the infiltration concerned all horses from the study population, the sum is not equal to 100%.

**Table 4:** Table showing the type of cellular infiltration in the submucosa of the rectum, along with its degree number of horses (*n* = 17) and % distribution in the studied horse population.

Infiltration Degree	Lymphocytes	Plasma Cells	Neutrophils	Eosinophils
mild	2 (11.8%) *	-	5 (29.4%)	9 (52.9%) *
moderate	10 (58.8%)	10 (58.8%)	-	3 (17.6%)
marked	2 (11.8%)	2 (11.8%)	-	2 (11.8%)
SUM	14 (82.4%)	12 (70.6)	5 (29.4%)	14 (82.4%)

* Perivascular infiltration in three horses. Note: A significant number of horses showed infiltration composed of more than one type of cell, so, although the infiltration concerned all horses from the study population, the sum is not equal to 100%.

**Table 5:** Table showing the value of albumin and total protein.

	Duodenal Biopsy	Rectal Biopsy	*p*
Albumin g/L (mean, SD)	30.6 (4.42)	22.7 (9.60)	*p* = 0.053
Total protein g/L (mean, SD)	63.6 (7.58)	54.1 (12.87)	*p* = 0.044
*n*	22	7	nd

## Data Availability

None of the data or models were deposited in an official repository. The data that support the findings of this study are available from the corresponding author upon reasonable request.
